# Fever Is Associated with Reduced, Hypothermia with Increased Mortality in Septic Patients: A Meta-Analysis of Clinical Trials

**DOI:** 10.1371/journal.pone.0170152

**Published:** 2017-01-12

**Authors:** Zoltan Rumbus, Robert Matics, Peter Hegyi, Csaba Zsiboras, Imre Szabo, Anita Illes, Erika Petervari, Marta Balasko, Katalin Marta, Alexandra Miko, Andrea Parniczky, Judit Tenk, Ildiko Rostas, Margit Solymar, Andras Garami

**Affiliations:** 1 Institute for Translational Medicine, Medical School, University of Pecs, Pecs, Hungary; 2 Department of Translational Medicine, First Department of Medicine, University of Pecs, Pecs, Hungary; 3 Momentum Gastroenterology Multidisciplinary Research Group, Hungarian Academy of Sciences - University of Szeged, Szeged, Hungary; 4 Department of Gastroenterology, First Department of Medicine, University of Pecs, Pecs, Hungary; Azienda Ospedaliero Universitaria Careggi, ITALY

## Abstract

**Background:**

Sepsis is usually accompanied by changes of body temperature (T_b_), but whether fever and hypothermia predict mortality equally or differently is not fully clarified. We aimed to find an association between T_b_ and mortality in septic patients with meta-analysis of clinical trials.

**Methods:**

We searched the PubMed, EMBASE, and Cochrane Controlled Trials Registry databases (from inception to February 2016). Human studies reporting T_b_ and mortality of patients with sepsis were included in the analyses. Average T_b_ with SEM and mortality rate of septic patient groups were extracted by two authors independently.

**Results:**

Forty-two studies reported T_b_ and mortality ratios in septic patients (*n* = 10,834). Pearson correlation analysis revealed weak negative linear correlation (*R*^2^ = 0.2794) between T_b_ and mortality. With forest plot analysis, we found a 22.2% (CI, 19.2–25.5) mortality rate in septic patients with fever (T_b_ > 38.0°C), which was higher, 31.2% (CI, 25.7–37.3), in normothermic patients, and it was the highest, 47.3% (CI, 38.9–55.7), in hypothermic patients (T_b_ < 36.0°C). Meta-regression analysis showed strong negative linear correlation between T_b_ and mortality rate (regression coefficient: -0.4318; *P* < 0.001). Mean T_b_ of the patients was higher in the lowest mortality quartile than in the highest: 38.1°C (CI, 37.9–38.4) vs 37.1°C (CI, 36.7–37.4).

**Conclusions:**

Deep T_b_ shows negative correlation with the clinical outcome in sepsis. Fever predicts lower, while hypothermia higher mortality rates compared with normal T_b_. Septic patients with the lowest (< 25%) chance of mortality have higher T_b_ than those with the highest chance (> 75%).

## Introduction

Sepsis constitutes a global burden for medical care with an estimated 31 million cases per year worldwide [1]. The incidence of sepsis has remained considerable [2–4] and it is associated with high mortality rates even nowadays [3]. It also underlies the importance and actuality of the topic for clinical praxis that the definitions of sepsis and associated illnesses have been updated recently [5].

As a systemic inflammation response, sepsis is often associated with changes of deep body temperature (T_b_), which can be manifested as fever or hypothermia in experimental animals [6–8], as well as in human patients [8–10]. Not surprisingly, deep T_b_ is regularly measured as one of the vital signs in the clinical praxis. In fact, many scoring systems (e.g., APACHE II, PIRO, SAPS II, SIRS), which help in the diagnosis or in the assessment of the progress of sepsis, include an abnormal deviation of T_b_ from the normal range [5, 11–14]. Usually T_b_s below 36.0°C or above 38.0°C are considered equally pathological [15], which values are in accordance with the criteria of the systemic inflammatory response syndrome [5, 11]. Based mainly on experimental data from animal studies Romanovksy and colleagues [6, 8] proposed that fever and hypothermia can both develop as two distinct adaptive mechanisms in sickness syndrome. The former characteristically occurs at the onset of an infection, representing an active fight against the pathogen, while the latter is usually associated with progressed stage or severity of the disease and it aims to secure the vital systems of the host [6, 8]. The two adaptive strategies can develop sequentially (e.g., early phase fever followed by late phase hypothermia) as the severity of the disease progresses [8], but hypothermia can be also one of the earliest developing events in animal models of endotoxin shock [16], moreover, septic patients admitted to ICU develop hypothermia more frequently in the early than in the late stages of their stay [17]. Despite the different pathological background of fever and hypothermia in systemic inflammation, both the increase and the decrease of T_b_ are evaluated commonly as equally severe signs in the clinical praxis [15]. This can be, at least in part, due to the standpoint that fever and hypothermia both represent an adaptive (though different) biological response to infection [8]. Accordingly, beneficial effects have been shown for elevated T_b_ on the clinical outcome of sepsis in clinical trials [18, 19], although no association between fever and disease severity was also reported [20]. Therapeutic (i.e., induced) hypothermia has been also shown to improve the outcome of sepsis in human studies [21, 22], but in case of spontaneously occurring hypothermia usually a positive association with mortality rate was found [20, 23, 24]. The definite association of T_b_ and mortality rate in a large study population has remained unknown.

We hypothesized that the deviation of T_b_ from the normal range predicts the clinical outcome in sepsis differently, and consequently septic patients with fever have lower chances for mortality than those who develop hypothermia. We performed an extensive literature search for human studies in septic patients and collected data on their T_b_ and mortality rate. The data were then analyzed with multiple statistical approaches, including Pearson regression, forest plot, and meta-regression analyses. Based on a high number of patients, we show a strong association between T_b_ and mortality ratio in sepsis across a wide temperature range.

## Materials and Methods

Our meta-analysis was conducted in accordance with the guidelines of the Preferred Reporting Items for Systematic Reviews and Meta-Analysis Protocols [25] (S1 Table). The analysis was based on the Participants, Intervention (prognostic factor), Comparison, Outcome (PICO) model: in septic population, we aimed to assess the predictive role of T_b_ deviations on the mortality ratio. No review protocol has been registered for the current meta-analysis.

### Search strategy

A search of the PubMed, EMBASE, and Cochrane Controlled Trials Registry databases was performed with using the following Medical Subject Headings and search terms (from inception to February 2016): sepsis OR bacteremia OR "septic syndrome" AND ("body temperature" OR fever OR hypothermia OR normothermia OR hyperthermia) AND (mortality OR survival). We restricted our search to original human studies published in English without time period limitations. Publications reporting immunosuppressive conditions (e.g., cancer, transplantation, HIV infection) were not included in the analysis. As a specific example, in the EMBASE database, which identified the highest number of articles, the term “sepsis OR bacteremia OR "septic syndrome" AND ("body temperature" OR fever OR hypothermia OR normothermia OR hyperthermia) AND (mortality OR survival) NOT (cancer OR immunosuppressive OR aids OR hiv OR transplantation)” was entered, and then the following filters were selected: humans, English, article, article in press, conference abstract, conference paper, major clinical study, case control study, clinical trial, cohort analysis, comparative study, controlled clinical trial, controlled study, cross-sectional study, double blind procedure, medical record review, multicenter study, observational study, outcomes research, phase 3 clinical trial, prospective study, randomized controlled trial, retrospective study. The search was conducted separately by two authors (ZR, AG), who also assessed study eligibility and extracted data from the selected studies independently. Disagreements were resolved by consensus with the help of a third party (MR).

### Study selection and data extraction

The titles and abstracts of the publications from the literature search were screened and the full text of potentially eligible articles was obtained. We included studies in which both the T_b_ values and the mortality ratios were reported for the same group(s) of patients with systemic inflammation accompanied by suspected or confirmed blood infection. From all included articles we extracted the sample size, the reported mean T_b_ value of the patients with its standard error (SEM), and the mortality ratio within the group during 28–30 days in most cases. To analyze the influence of fever, normothermia, and hypothermia on the mortality ratio in sepsis we separated the collected data into three study groups based on the mean T_b_ of the patients.

### Statistical analysis

We have used event rates (mortality rates) as effect size data. Studies were grouped by T_b_ as low (up to 36.0°C; *n* = 890), medium (36.1 to 38.0°C; *n* = 3,904) and high (above 38.0°C; *n* = 6,040) and forest plots in the three groups were used to describe mortality. Selection of the T_b_ groups was based on the SIRS criteria [5, 11]. Another grouping was conducted by mortalities, these were split into quartiles and the means of T_b_s were compared by investigating the presence or absence of overlaps in the 95% confidence intervals (CI), just like in case of the grouping by T_b_.

Between-study heterogeneity was tested with *Q* homogeneity test (*P* values of less than 0.05 were considered as indicators of significant heterogeneity) and with *I*^2^ statistical test, where *I*^2^ is the proportion of total variation attributable to between-study variability (an *I*^2^ value of more than 50 was considered as indicating considerable heterogeneity). These two values were used to model selection purposes as well (fixed vs random). The tests revealed considerable heterogeneity in the overall study population (*Q* = 809.509; *I*^2^ = 89.25) and also in all three T_b_ groups, in particular *Q* = 270.447; *I*^2^ = 85.58 in the high, *Q* = 373.357; *I*^2^ = 90.63 in the medium, and *Q* = 36.843; *I*^2^ = 70.14 in the low T_b_ group. Consequently, we applied the random effect model in our forest plot and meta-regression analyses.

Publication bias was tested by inspecting the funnel plot. Meta-regression was performed to assess the overall effect of T_b_ to mortality. Except for the Pearson correlation analysis for which Microsoft Excel 2010 (Microsoft Corporation, Redmond, WA, USA) was used, all analyses were performed with the Comprehensive Meta-Analysis software (Biostat, Inc., Engelwood, MJ, USA).

## Results

### Study selection

**Fig 1:**
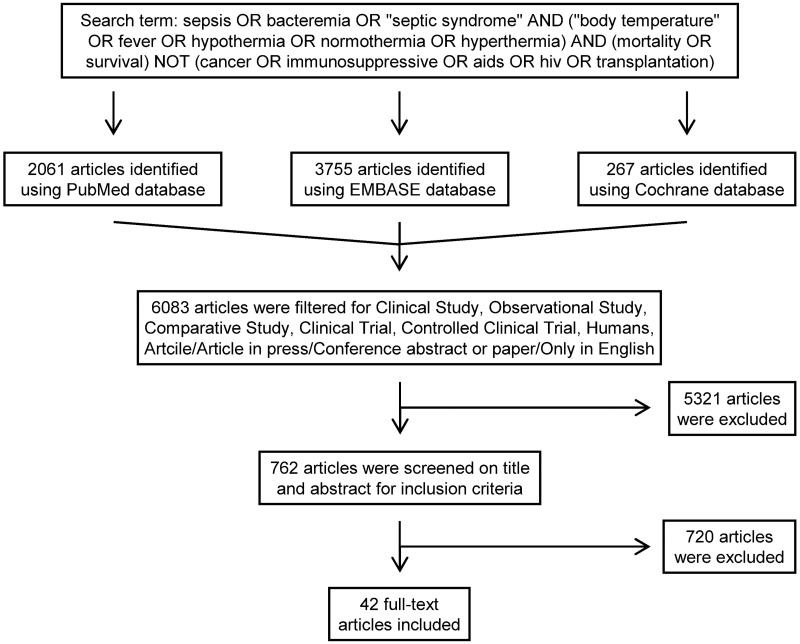
Flowchart of study selection and inclusion.

The flow chart of the study selection is presented in Fig 1. Until February 29, 2016 the electronic literature search identified altogether 6,083 studies from the PubMed, EMBASE, and Cochrane databases. After enabling filters for human studies and English language, 762 articles remained, which were screened on title and abstract for inclusion criteria. In 720 studies T_b_ or mortality rate was not suitably reported in the septic patients, these were also excluded, as a result 42 full-text publications were found eligible for statistical analysis which included data from a total of 10,834 septic patients.

### Incidence of mortality in septic patients with fever, normothermia, and hypothermia

As a rude approach, first we performed a common (Pearson) correlation analysis between T_b_ and mortality rate of all septic patients. A weak negative linear correlation was found (y = -0.0909x + 3.6902; *R*^2^ = 0.2794), which suggests an association between T_b_ and mortality in sepsis. This method, however, did not allow us to weight the collected data according to the size of the studied populations, thus a detailed meta-analysis was needed.

**Fig 2:**
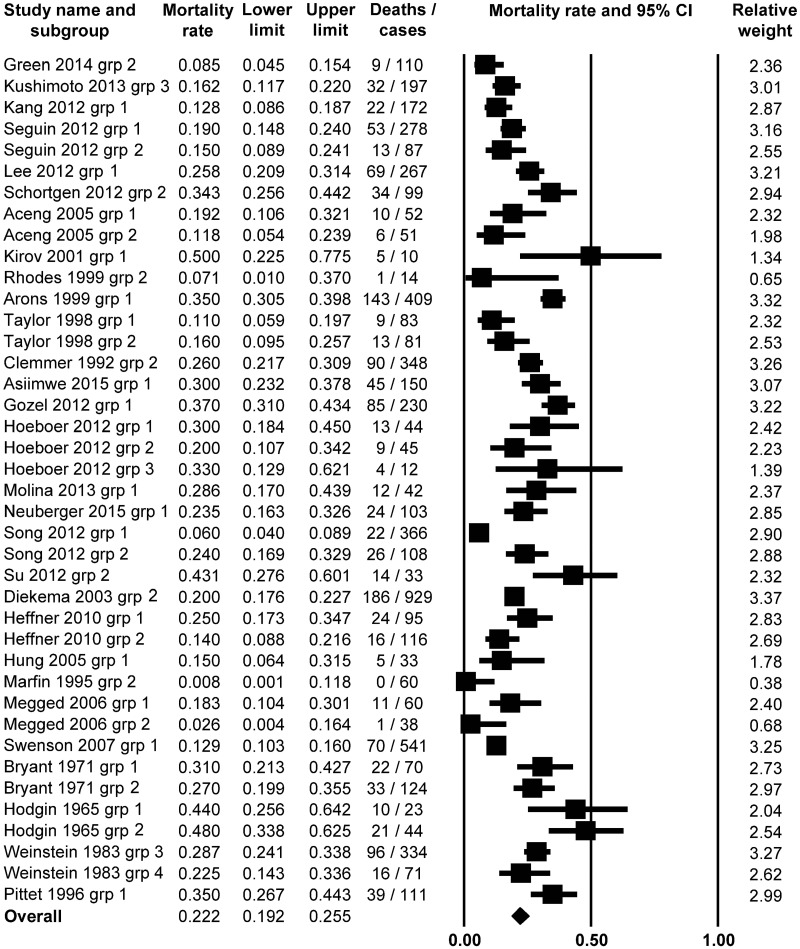
Forest plot analysis of mortality rate using random-effects model in septic patients with fever (body temperature above 38.0°C; *n* = 6,040).

First, we investigated the incidence of mortality in fever associated with sepsis. We found 29 studies [18–20, 23, 26–50], in which the authors reported fever (defined as T_b_ > 38.0°C) in sepsis. From these studies, 40 groups of septic patients could be separated and included in the analysis with the random effect model. The meta-analysis of the mortality rates in the septic patients with fever revealed an average event rate of 22.2% (95% CI, 19.2–25.5%; Z = -13.4331) (Fig 2). This percentage was significantly (*P* = 0.000) lower than the 50% chance of mortality, which could be regarded as a random outcome.

**Fig 3:**
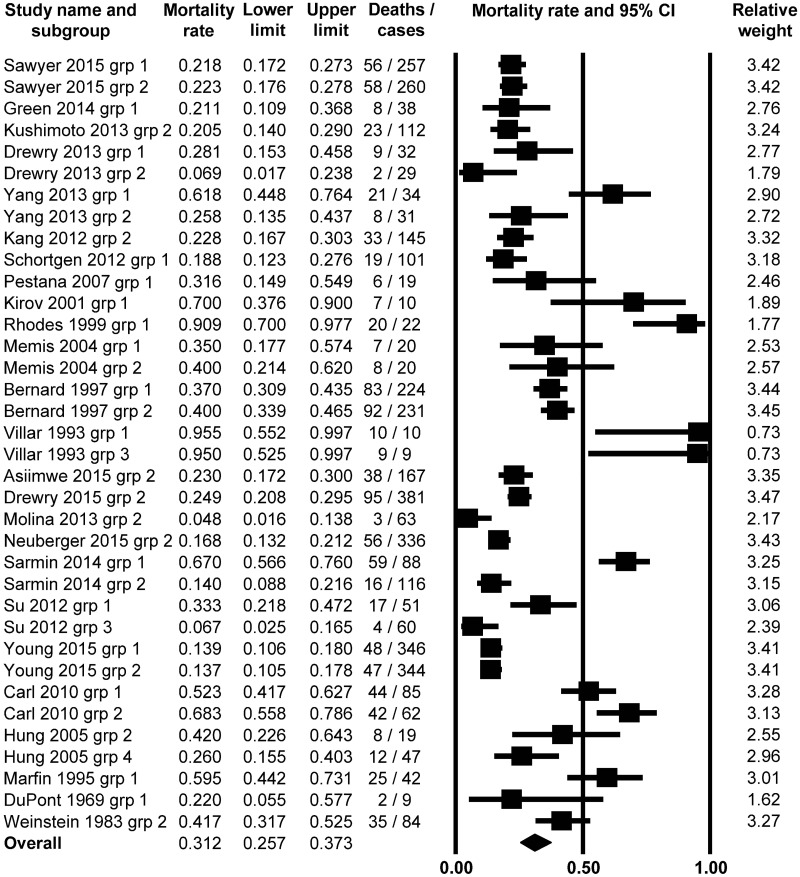
Forest plot analysis of mortality rate using random-effects model in septic patients with normothermia (body temperature between 36.1 and 38.0°C; *n* = 3,904).

Next, we analyzed the mortality ratios of patients who developed neither fever nor hypothermia in association with sepsis, therefore this population could be regarded as normothermic (T_b_ = 36.0–38.0°C). From the 25 studies, in which normal T_b_ was reported in the septic patients [20, 22, 24, 28, 31, 35–37, 39–41, 43, 44, 47, 50–60], 36 subgroups of patients were separated, which were then analyzed with the random effect model. We found that the average mortality ratio was 31.2% (95% CI, 25.7–37.3%; Z = -5.7089) (Fig 3), which was higher than in the fever group. The mortality rate was significantly (*P* < 0.001) lower than 50% in this study population.

**Fig 4:**
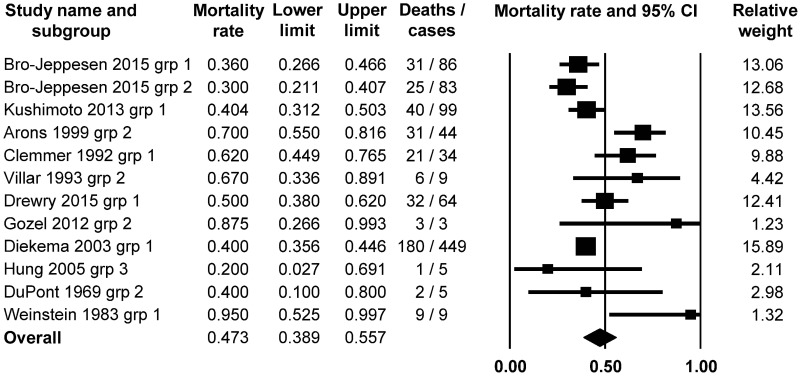
Forest plot analysis of mortality rate using random-effects model in septic patients with hypothermia (body temperature up to 36.0°C; *n* = 890).

Then, we examined the incidence of mortality in hypothermic (T_b_ < 36.0°C) septic patients. We identified 11 studies [20, 22–24, 27, 29, 30, 35, 50, 54, 61], which included data on both T_b_ and mortality in septic patients. From these, the patients could be divided in 12 subgroups, which served as the basis of the meta-analysis. The random effect model revealed that the average mortality rate was the highest, 47.3% (95% CI, 38.9–55.7; Z = 0.520491) in the hypothermic patients (Fig 4), which did not significantly differ from the 50% random chance (*P* = 0.603).

**Fig 5:**
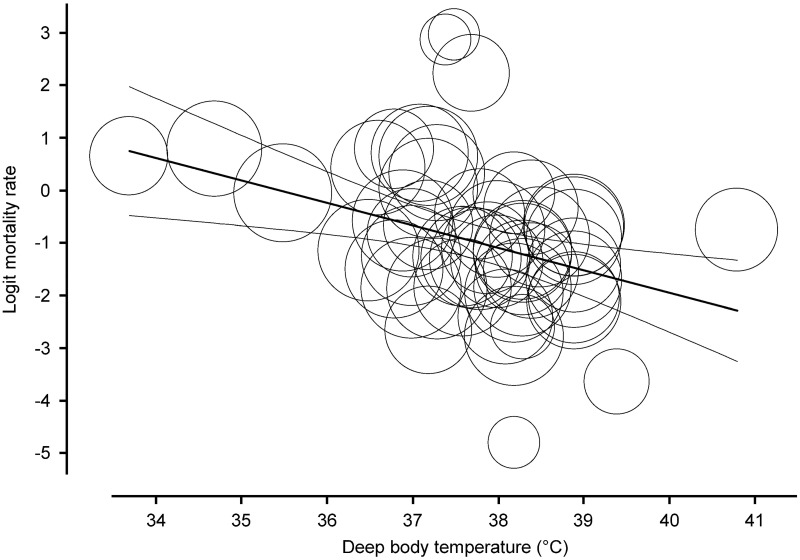
Meta-regression analysis of the association between body temperature and mortality ratio in septic patients (*n* = 10, 834).

As a further statistical approach, we also performed a meta-regression analysis on the collected data. We found a significant (*P* < 0.001) negative linear correlation between T_b_ and mortality rate (regression coefficient: -0.4318; 95% CI, -0.6699 - -0.1938) based on 51 studies included in the analysis (Fig 5).

Last, we divided the patients into quartiles (Q1-Q4) of mortality ratios (Q1: 0–25, Q2: 26–50, Q3: 51–75, and Q4: 76–100%) and calculated the average T_b_ for each mortality quartile. The weighted average T_b_s were 38.1 (95% CI, 37.9–38.4°C), 37.8 (95% CI, 37.5–38.2°C), 37.6 (95% CI, 36.5–38.7°C), and 37.1°C (95% CI, 36.7–37.4°C) in the Q1, Q2, Q3, and Q4 groups, respectively. These results also indicate that in sepsis a higher T_b_ is associated with better outcome, while a lower T_b_ is related with higher risk of mortality. Of note, the T_b_s in Q1 and Q4 (i.e., in the groups with lowest and highest mortality, respectively) are clearly distinct from each other, as the 95% CIs do not overlap.

## Discussion

In the current analysis we revealed a clear association between T_b_ and mortality in septic patients by using a detailed statistical approach which was based on an extensive literature search of previous human studies. We found that the presence of fever reduces, while that of hypothermia promotes mortality in septic patients as compared to normothermic subjects.

From previous studies of septic patients only limited information is available to show an association between T_b_ and mortality in a wide temperature range. While a worse outcome was consistently found to be related to hypothermia [20, 23, 24, 27], those studies compared hypothermia with fever [23, 27] or with nonhypothermic (i.e., by merging febrile and normothermic) patients [20, 24]. In one study, no association was found between hypothermia and the clinical outcome of sepsis [19]. Regarding the role of fever, different clinical trials came to controversial results in sepsis. Several authors showed that a higher T_b_ was beneficial [18, 19, 50, 62], others that it was disadvantageous [44, 63], and a few found no association between fever and mortality [20, 60]. The discrepancy among the studies may result from the limited sample size used in the trials. Another explanation for not detecting significant association could be the insensitivity of the used statistical method. Indeed, when we first performed a commonly used regression analysis, the Pearson correlation, which did not allow us to weight the collected data for example for sample size, we found only a very weak negative correlation (*P* = 0.047) between T_b_ and mortality ratio in sepsis. Thus, we applied more precise statistical tools (forest plot and meta-regression analyses).

In our analyses, we used a sizeable, heterogeneous population of septic patients (*n* = 10,834) with a wide range of T_b_ (33.0–39.9°C). We showed that in sepsis mortality rates are lower if fever is present and higher in cases of hypothermia as compared to the normothermic group. In addition, we demonstrated a strong negative correlation (*P* = 0.0004) between T_b_ and mortality ratio with the help of meta-regression analysis. In our statistical approach, we also included a substantial number of septic patient groups with average T_b_s within the normal range (*n* = 3,904). Furthermore, when we calculated the mean T_b_s of septic patients in the mortality quartiles, we found that it gradually decreased from the lowest to the highest quartile and it was significantly higher in the lowest (0–25%) than in the highest quartile (75–100%) of mortality (38.1 ± 0.1 vs 37.1 ± 0.2°C for mean ± SEM, respectively). Taken together the results from all of our statistical approaches, our data strongly suggest a predictive role of T_b_ for the outcome of sepsis.

Sepsis continues to constitute a major challenge in critical care medicine [1–4]. As a systemic inflammation process, sepsis is frequently accompanied by abnormalities of T_b_, like fever and hypothermia. In animal experiments, lower doses of endotoxin usually cause fever, whilst higher doses lead to the development of hypothermia [64, 65], indicating that the severity of the disease determines the change in T_b_ and not the way around. Based on our statistical analyses of human studies fever seems beneficial, but hypothermia rather disadvantageous for the organism regarding the outcome. However, it has to be noted that the current analysis does not allow us to conclude that the change of T_b_ per se is responsible for the lower and higher mortality rates in fever and hypothermia, respectively. Instead of a cause-effect relationship, the abnormal T_b_ should be rather regarded as a prognostic vital parameter of the severity and progress of the inflammation, and as such, as a warning sign, which can help doctors to asses the outcome of to the infection.

With regard to the adaptive biological value of T_b_ alterations in mammals, the development of fever in systemic inflammation is considered to indicate the activation of defense mechanisms of the body to fight the intruding agent [6–8]. By enhancing immune functions and accelerating the elimination of the microorganism from the body, at the onset of the inflammation fever is an adaptive, beneficial thermoregulatory response, although it involves a higher energy cost [6–8]. Therefore, fever itself is assumed to have a direct, advantageous effect on the mortality ratio in systemic inflammation (e.g., sepsis), when it is affordable for the host. However, T_b_ regulation should be considered in the framework of complex energy balance [66], therefore, the beneficial value of fever as an energetically expensive defense response is doubtable when there is a risk of energy deficiency, which often develops as the severity of the disease further progresses. In support of that, the administration of antipyretics resulted in an increase of mortality rate of critically ill patients in prospective clinical trials [38, 67]. However, in severe sepsis or septic shock, the use of pharmacological antipyretics did not influence mortality [51, 68], while fever control with external cooling decreased early mortality in human studies [44].

Spontaneous hypothermia represents a distinct, adaptive mechanism to systemic inflammation in experimental animals [69] and in septic patients [17]. It characteristically develops in severe cases of already progressed diseases, when—instead of actively coping with the microorganism—the organism attempts to increase survival by saving its energy resources [6, 8]. A recent study by Fonseca et al. [17] revealed that spontaneous hypothermia is a transient, self-limiting, and nonterminal event in human sepsis, which underlies its biological value as an adaptive mechanism in the critically ill patients.

Although the results of our analysis showed that hypothermia is associated with higher mortality, it should be noted that we can not be sure how mortality ratio of the patients would have changed if hypothermia had not developed or if the patients were rewarmed. As of today, to our knowledge, the effect of rewarming vs non-rewarming on the mortality of septic patients with spontaneous hypothermia has not been compared in randomized controlled trials. Therefore, hypothermia in itself should not be regarded harmful for the body as the associated higher mortality rate of the septic patients is presumably due to their more severe clinical condition. We suggest that the difference between the mortality rates of febrile and hypothermic patients with sepsis is due to the different severity and progression of the inflammation and not due to T_b_ itself. As a consequence, T_b_ itself serves not as a detrimental factor, but instead, as an indicative predictor for the severity of the disease and as such for mortality in sepsis.

From a clinical perspective, our results highlight the importance of precise and regular measurements of deep T_b_, since its abnormalities can help physicians—especially in critical care medicine—not only in the diagnosis, but also in the follow up of the progression, and in the prognosis of sepsis. Based on our findings, it would be worth to consider that hypothermia should be weighted differently than fever and not equally as currently used in many scoring systems (e.g., SIRS, APACHE, PIRO), since hypothermia indicates a more severe stage of sepsis, and, therefore it is associated with worse clinical outcome. Regarding therapeutic interventions, the T_b_ management of septic patients should be always carefully evaluated and perhaps guidelines could be established (e.g., for the initiation of antipyretic treatment) to improve the clinical outcome in sepsis.

## Conclusions

The abnormalities of deep T_b_ are strongly associated with the clinical outcome in sepsis. The mortality ratio of febrile patients is lower, while in patients with hypothermia it is markedly higher than that of patients with normal T_b_. In cases of sepsis, there is a strong negative correlation between the mortality ratio and deep T_b_ in a wide temperature range. Septic patients with the lowest (< 25%) chance of mortality have significantly higher deep T_b_ than those who belong to the highest mortality quartile (> 75%).

## Strengths and Limitations

Our meta-analysis included data from a total of 10,834 septic patients with overall 2,724 mortality events. We believe that our search strategy was adequately broad and included the three main databases of human studies. As result, 42 full-text articles could be indentified and used in our analyses. Although the sample size and the overall event rate can be considered large enough to draw solid conclusions about the association of T_b_ and mortality rate in sepsis, our study has certain limitations.

First, due to the nature of the meta-analysis method, we have studied the reported mean T_b_s in populations of patients, rather than the association between T_b_ and the outcome of sepsis in individual patients. The latter approach would certainly allow one to draw firmer conclusions about the association between T_b_ and mortality, but it would also necessitate access to the original data of the analyzed articles, which is not feasible. Alternatively, a well-designed clinical trial with a big sample size could also provide high-quality individual data and based on our results it can be warranted to conduct such trials.

Second, the studied population of patients is quite diverse, which diversity could also have its own impact on the results. For example, T_b_ measurements were performed in different ways and not at the same time points in the analyzed studies. Despite such differences, we believe that the size of the analyzed sample was big enough to mitigate the methodological differences among the studies and to allow for drawing conclusions about the association of T_b_ and mortality in the septic patients.

## Supporting Information

S1 Table PRISMA 2009 checklist. (DOC)
